# Longitudinal analysis of genomic and immune differences between primary and metastatic nasopharyngeal carcinoma for precision oncology

**DOI:** 10.3389/fonc.2026.1897657

**Published:** 2026-08-27

**Authors:** Ying Lu, Tiantian Han, Lihui Wang, Xishan Chen, Huan Lin, Mengxiao Wang, Dongsheng Chen, Haixin Huang

**Affiliations:** 1 Department of Oncology, The Fourth Affiliated Hospital of Guangxi Medical University, Liuzhou, China; 2 State Key Laboratory of Neurology and Oncology Drug Development, Jiangsu Simcere Diagnostics Co., Ltd., Nanjing Simcere Medical Laboratory Science Co., Ltd., Nanjing, China

**Keywords:** distant metastasis, genomic profiling, nasopharyngeal carcinoma, precision treatment, tumor immune microenvironment

## Abstract

**Introduction:**

Nasopharyngeal carcinoma (NPC) is a common malignancy with a high incidence in Southern China and Southeast Asia. Distant metastasis remains a major cause of poor prognosis. This study aims to explore the genomic and immune microenvironmental changes in primary and metastatic NPC through longitudinal analysis, to provide insights for guiding precision treatment strategies.

**Methods:**

We analyzed tumor samples from 11 male NPC patients with distant metastasis. Paired primary and metastatic samples underwent targeted whole-exome sequencing (551 genes) and RNA sequencing (289 genes). Multiplex immunohistochemistry was performed to assess immune cell composition and immune response markers. Genomic alterations and immune features were compared between primary and metastatic tumors, and their associations with clinical outcomes were evaluated.

**Results:**

Metastatic tumors exhibited distinct chromosomal changes, including frequent 1q gain, while 6p gain showed a trend toward prolonged survival. Primary tumors showed stronger immune suppression characterized by increased B-cell and Treg infiltration and elevated CTLA4 and IDO1 expression, whereas metastatic lesions displayed more active immune responses. Liver metastases presented a distinct immune landscape with lower CD8^+^ T-cell density compared to non-liver metastases. In locoregionally advanced NPC, high expression of oncogenic genes such as EGFR and MYC correlated with shorter disease-free survival, while elevated PANCK expression and enhanced cytotoxicity were linked to better outcomes. These results delineate the molecular and immune evolution of NPC and provide a foundation for developing precision therapeutic strategies.

**Conclusion:**

This study identifies key molecular and immune features associated with NPC survival. Primary tumors show an immunosuppressive phenotype, suggesting potential benefits from combining CTLA4 or IDO1 inhibitors with PD-1 blockade. Liver metastases exhibit distinct immune features, supporting the need for site-specific precision therapies. These findings contribute to personalized management strategies in NPC, guiding treatment based on molecular and immune profiles.

## Highlights:

1. Longitudinal multi-omics analysis reveals genomic alterations linked to NPC metastasis
2. Primary tumors show stronger immune suppression and site-specific immune diversity
3. Combined CTLA4 or IDO1 and PD-1 inhibition may offer precision immunotherapy benefits

## Introduction

Nasopharyngeal carcinoma (NPC) is a common malignancy of the head and neck, with particularly high incidence in Southern China and Southeast Asia, much higher than that in Western countries (1). Histologically, NPC is classified into keratinizing squamous cell carcinoma, non-keratinizing carcinoma (including differentiated and undifferentiated types), and basaloid squamous cell carcinoma (2). The non-keratinizing subtype is the endemic form of NPC and is mainly observed in Asian populations (3, 4). Most cases of non-keratinizing NPC are associated with Epstein–Barr virus (EBV) infection (1). EBV infection, genetic susceptibility, and environmental factors together drive the development of NPC (5).

The clinical presentation of NPC is often insidious, and more than 70% of patients are diagnosed at a locally advanced stage (6). According to stage, patients may receive radiotherapy, chemotherapy, targeted therapy, or immunotherapy, either alone or in combination. The 5-year survival rate is approximately 85% (7). However, 20%–30% of patients develop locoregional recurrence and/or distant metastasis after treatment (8), and 4%–10% already present with distant metastasis (DM) at the time of initial diagnosis (9). Distant metastasis remains the major cause of poor outcomes. The most frequent metastatic sites include bone, lung, liver, and distant lymph nodes, and once metastasis occurs, prognosis is usually unfavorable (10). Understanding the biological differences between primary and metastatic lesions is therefore important for improving clinical management of NPC.

In recent years, multi-omics studies have provided important insights into NPC biology. Whole-genome and exome sequencing have revealed recurrent abnormalities in the NF-κB signaling pathway, PI3K/AKT activation, chromatin remodeling, and DNA repair (11). Bruce et al. reported viral–host cooperation leading to NF-κB activation and immune escape in NPC (12). Other studies have suggested that alterations in KMT2D, CYLD, PIK3CA, and SF3B1 may be associated with metastatic behavior (13). In addition, some investigations have demonstrated that primary and metastatic tumors may differ at the genomic level, indicating that metastatic lesions are not simply identical to their primaries (14). However, most existing studies have focused separately on either primary or metastatic NPC, with limited systematic analyses of paired primary and metastatic lesions.

The tumor immune microenvironment (TIME) of NPC is characterized by abundant lymphocyte infiltration and high PD-L1 expression, suggesting potential sensitivity to immunotherapy. At the same time, immune tolerance, T-cell exhaustion, infiltration of myeloid suppressor cells, and stromal barriers limit treatment benefit (15, 16). Although previous studies have confirmed that the TIME plays an important role in treatment response and outcomes, systematic comparisons between the immune features of primary and metastatic lesions are still lacking.

Based on this background, we analyzed paired and unpaired samples of primary and metastatic NPC. By integrating genomic, transcriptomic, and multiplex immunohistochemistry (mIHC) data, we aimed to characterize the molecular and immune microenvironmental differences across lesions and to explore their associations with clinical outcomes. Our goal was to generate clinically relevant evidence that could support stratified management and optimize treatment strategies for NPC.

## Methods

### Patient cohort and sample collection

Paraffin tissue samples of primary lesions and metastatic lesions from 8 patients with metachronous metastatic NPC and 3 patients with synchronous NPC admitted to our hospital from August 2019 to August 2024 were retrospectively collected. The use of these tissue samples for this study was approved by the hospital ethics committee (Approval No. KY2022054). As this study adopted a retrospective design, informed consent was waived.

### DNA extraction and library preparation

Genomic DNA (gDNA) was extracted from formalin-fixed paraffin-embedded (FFPE) tumor tissues or frozen fresh tumor samples using the Genomic DNA Tissue Extraction Kit (Concert^®^). The extracted gDNA was quantified using the Qubit dsDNA HS Assay Kit with a Qubit Fluorometer (Thermo Fisher Scientific) and assessed for quality using an Agilent 4200 TapeStation (Agilent).

For library preparation, 200 ng of qualified gDNA was enzymatically fragmented into 200–300 bp fragments. DNA end repair, A-tailing, and adaptor ligation were performed using the KAPA Hyper DNA Library Prep Kit (Roche Diagnostics) and the VAHTSTM Universal DNA Library Prep Kit for Illumina^®^ (Vazyme). Unligated adaptors were removed by size selection with Agencourt AMPure XP beads (Beckman Coulter). The ligation products were amplified via PCR to produce a pre-library for hybridization.

Sequencing of the prepared libraries was performed using 150-bp paired-end reads on the Illumina NovaSeq 6000 platform. All procedures were conducted according to the manufacturer’s protocols at the CAP-accredited central laboratory of Jiangsu Simcere Diagnostics Co., Ltd. (Nanjing, China).

### Bioinformatics analysis

Sequencing raw data were processed to generate FASTQ files, followed by quality control using fastp (v2.20.0) for adapter trimming and removal of low-quality reads. Clean reads were aligned to the human reference genome (hg19/GRCh37) using the BWA-MEM algorithm (v0.7.17). Somatic single nucleotide variations (SNVs) and insertions/deletions (Indels) were identified and annotated using VarDict (v1.5.7) and InterVar, respectively. Variants were filtered against public databases, including the 1000 Genomes Project and Exome Aggregation Consortium Browser, to exclude common polymorphisms. Copy number variations (CNVs) were analyzed using CNVkit, while fusions were detected with Factera (v1.4.4).

Tumor mutation burden (TMB) was calculated by summing all nonsynonymous somatic mutations within the coding region per megabase, excluding known somatic mutations listed in COSMIC. Microsatellite instability (MSI) was determined using a custom pipeline. The MSI score was calculated as the percentage of unstable loci. Samples with MSI scores above the predefined threshold (≥0.15) were classified as MSI-high.

### Transcriptional profiling and analysis

Total RNA was extracted from FFPE samples using the Qiagen RNeasy FFPE Kit and quantified with a NanoDrop ND1000 spectrophotometer (Thermo Fisher Scientific). RNA samples (100 ng) were hybridized overnight at 65 °C to a customized NanoString PanCancer code set targeting 289 genes associated with immune regulation, tumor biology, and the tumor microenvironment. Hybridized samples were processed using the nCounter Prep Station, and gene expression data were obtained via the nCounter Digital Analyzer.

Housekeeping genes were used to normalize the expression data using nSolver 2.6 software. Differentially expressed genes (DEGs) were identified with the DESeq2 package, with the cutoff: log2 |fold change| > 1 and false discovery rate (FDR) < 0.05. Volcano and heatmap plots of DEGs were generated using the ggplot2 and ComplexHeatmap packages, respectively. Gene set enrichment analysis (GSEA) was performed using Hallmark gene sets from the Molecular Signatures Database (MSigDB). metagene scores of 14 immune cell types (e.g., T cells, B cells, macrophages, NK cells) were calculated as the geometric mean of the expression levels of the constituent genes. the full list of the 289 immune-related genes, as well as the marker genes of each cell type are listed in **Table 1** Clinicopathological features of 11 patients with Nasopharyngeal carcinoma.

### Multiplexed immunofluorescence

Multiplex immunofluorescence staining was performed using the Leica Bond RX automated system, following the manufacturer’s protocol. The following primary antibodies were applied for staining: anti-CD68 (Ventana, KP-1, 1:100), anti-CD163 (Ventana, EPR19518, 1:100), and anti-PanCK (Ventana, AE1/AE3, 1:100), anti-CD80 (Abcam, ab134120, 1:100), anti-Ki67 (BD Biosciences, 563757, 1:100). Nuclei were counterstained with 4′,6-diamidino-2-phenylindole (DAPI; Sigma). Immunohistochemistry (IHC) was performed to evaluate the tumor microenvironment. The following primary antibodies were used: anti-PD-L1 (clone SP263, Ventana Medical Systems, Tucson, AZ, USA), anti-CD8 (clone SP57, Ventana Medical Systems), anti-FOXP3 (clone 236A/E7, Abcam, Cambridge, UK), anti-CD68 (clone KP-1, Ventana Medical Systems), and anti-CD163 (clone EPR19518, Abcam). To ensure staining consistency and mitigate any potential batch-to-batch variation of the antibodies, appropriate positive and negative tissue controls were included in every staining run.

### Statistical analysis

The statistical analyses and visualization were conducted using R 4.1.3. Survival data were analyzed with Kaplan–Meier curves, and the log-rank test was used for comparison. Group differences were evaluated using the Wilcoxon test, and a p-value of less than 0.05 was considered statistically significant.

## Result

### Patients with NPC and samples

**Figure 1:**
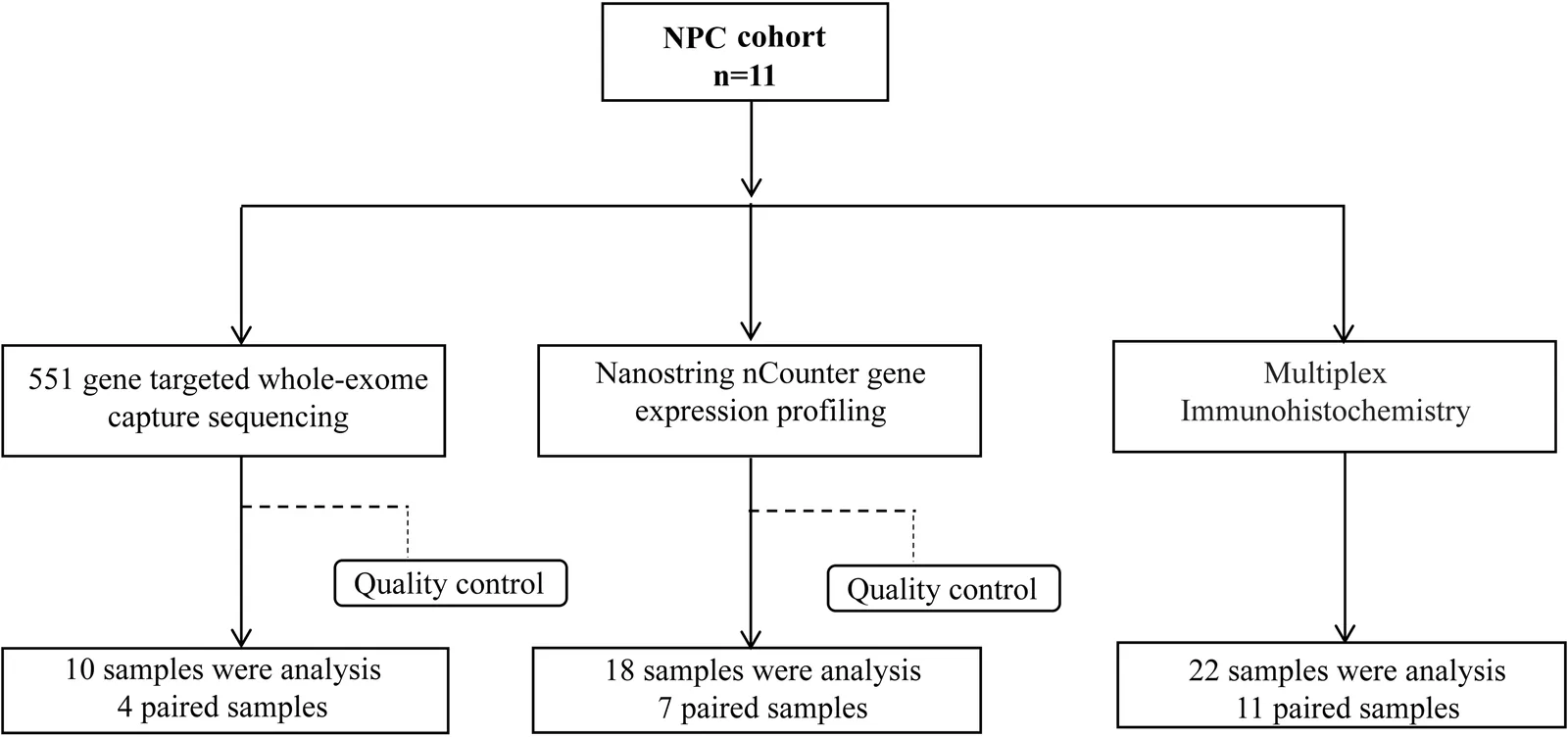
Study workflow of the nasopharyngeal carcinoma (NPC) cohort.

The clinicopathological features of the 11 nasopharyngeal carcinoma (NPC) patients with distant metastasis are summarized in **Table 1**. The mean patient age was 42 years, and all patients were male. A total of 22 tumor samples obtained from these 11 patients were subjected to targeted whole-exome sequencing of 551 genes using an NGS platform and RNA sequencing of 289 genes using a NanoString platform. After stringent quality control, 10 samples were included in the whole-exome sequencing analysis and 18 samples in the RNA sequencing analysis. In parallel, multiplex immunohistochemistry (mIHC) was performed on all 22 samples to evaluate five immune markers. The overall detection workflow is illustrated in **Figure 1**.

**Table 1:** Clinicopathological features of 11 patients with Nasopharyngeal carcinoma.

Patient	year	sex	lesion size	EBV DNA(copies/mL)	T stage	N stage	Metastatic location
P01	46	male	3mm	>500	3	2	Lung
P02	32	male	>5mm	>500	3	2	bone
P03	31	male	>5mm	>500	2	1	liver
P04	50	male	>5mm	< 500	4	1	Lung
P05	38	male	2mm	>500	3	2	Brain
P06	47	male	>5mm	>500	2	3	liver
P07	44	male	>5mm	< 500	3	2	liver
P08	44	male	>5mm	>500	3	2	liver
P09	32	male	>5mm	>500	4	2	liver
P10	51	male	4mm	–	3	2	Pelvic
P11	51	male	>5mm	>500	3	3	liver

### Genome differences between primary and metastatic NPC

**Figure 2:**
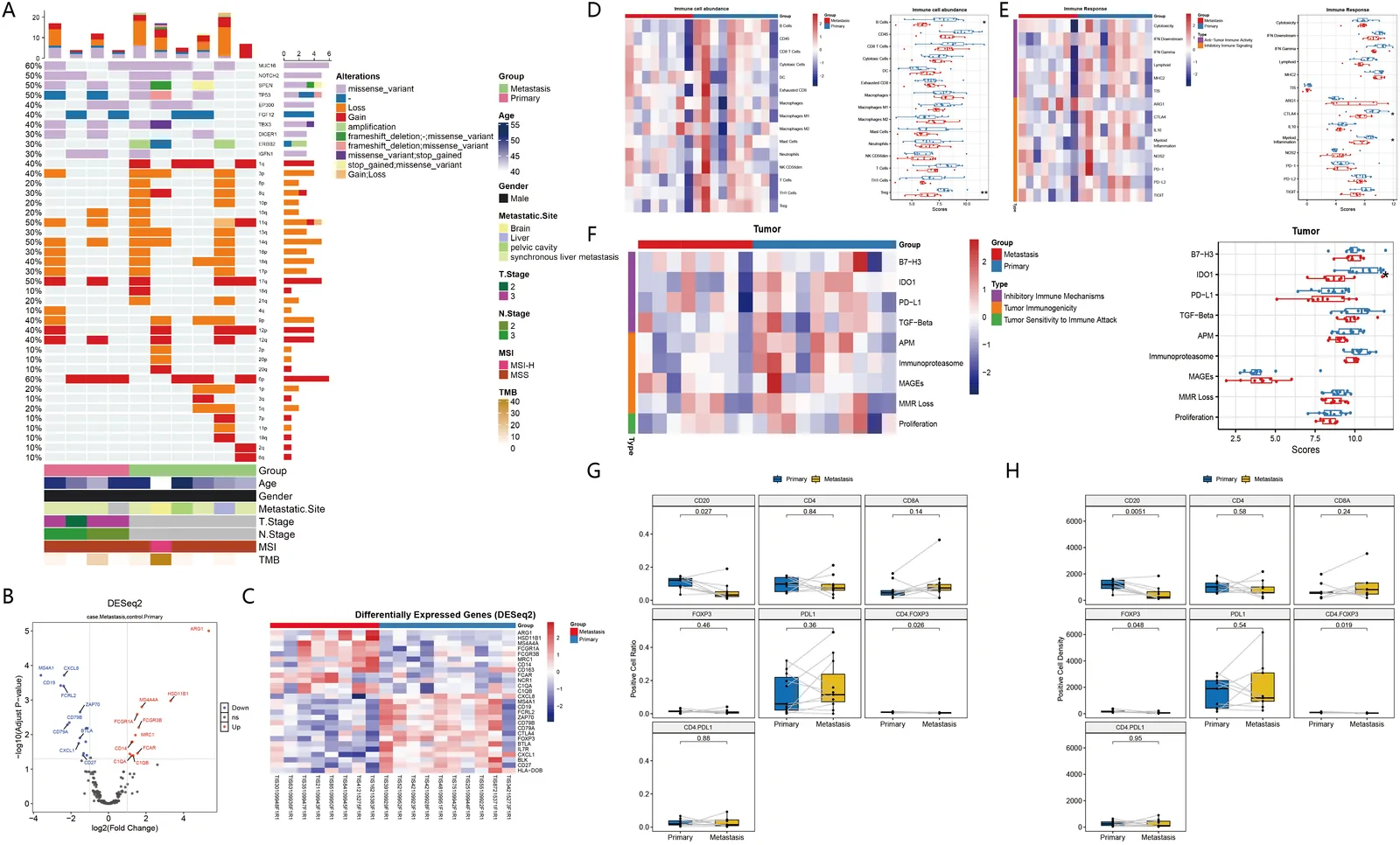
Integrated genomic, transcriptomic, and immune profiling of primary and metastatic NPC. **(A)** Landscape of genomic alterations identified by 551-gene targeted panel sequencing in 11 NPC patients with distant metastasis. The spectrum includes single-nucleotide variants (SNVs), copy number variations (CNVs), and insertions/deletions (indels). Metastatic tumors exhibited distinct genomic patterns with enrichment of chromosome 1q gain. **(B)** Volcano plot showing differentially expressed genes (DESeq2 analysis) between metastatic and primary tumors. **(C)** Heatmap of differentially expressed genes detected by NanoString nCounter RNA profiling, illustrating transcriptional differences between primary and metastatic lesions. **(D, E)** Heatmaps and box plots showing immune cell composition and immune response scores derived from NanoString immune profiling, highlighting altered immune landscapes between primary and metastatic tumors. **(F)** Expression heatmap and corresponding box plots of selected immune checkpoint and regulatory molecules.**(G, H)** Quantitative analysis of immune cell populations assessed by multiplex immunohistochemistry (mIHC) in paired primary and metastatic samples (n=11). The densities and proportions of CD20^+^B cells, CD4^+^T cells, CD8^+^T cells, FOXP3^+^regulatory T cells, and PD-L1^+^cells were compared using the Wilcoxon signed-rank test. *P < 0.05; **< 0.01.

To elucidate the genomic differences between primary and metastatic tumors, we compared the gene mutation profiles between primary and metastatic tumors (**Figure 2A**). Notably, the increase in chromosome arm 1q was significantly higher in metastatic tumors (**Figure 2B**), several chromosomal regions displayed significant alterations in metastatic tumors compared to primary tumors. Furthermore, survival analysis (**Figure 2C**) demonstrated that an increase in 6p was associated with an improved prognosis in patients with metastatic disease. Patients possessing 6p gain showed a trend toward prolonged survival compared with patients without this alteration, although the difference was not statistically significant (p = 0.3), potentially attributable to the modest sample size.

### TIME differences between primary and metastatic NPC

To investigate the TIME (tumor immune microenvironment) differences between primary and metastatic tumors, a comparative analysis of immune cell composition and immune response features was conducted. As shown in **Figure 2D**, primary tumors exhibited significantly higher abundance of B cells and regulatory T cells (Tregs) compared to metastatic samples. In terms of immune response characteristics, primary tumors showed elevated expression of CTLA4 and increased activity of myeloid inflammation-related pathways, as indicated by higher scores in CTLA4 and myeloid inflammation signatures (**Figure 2E**). These features suggest a more immunosuppressive microenvironment in primary tumors compared to metastatic tumors. In accordance with the immunosuppressive state of TIME in the primary tumors, we also found that IDO1 expression was significantly elevated in primary tumors compared to metastases (**Figure 2F**). This finding further suggests that primary tumors’ TIME has a distinct immunosuppressive profile. To characterize immune cell composition in the TIME of primary versus metastatic tumors, we performed multiplex immunohistochemistry (mIHC) to quantify both the proportions (**Figure 2G**) and densities (**Figure 2H**) of immune cell populations.

To characterize immune cell composition in the TIME of primary versus metastatic tumors, weperformed multiplex immunohistochemistry (mIHC) to quantify both the proportion and density of immune cells. A statistically significant discrepancy was observed in the ratio of B cells between primary and metastatic tumors, the ratio of B cells was significantly higher in primary tumors than in metastatic tumors (p = 0.027). Concurrently, the proportion of regulatory T cells (Tregs) exhibited a higher frequency in primary tumors (p = 0.026), suggesting increased Treg infiltration. The density analysis consistently revealed that primary tumors harbored significantly more B cells (p = 0.0051), Tregs (p = 0.048), and CD4+ Tregs (p = 0.019) compared to metastatic tumors. No significant differences were observed in the densities of CD8+ T cells, CD4+ T cells, or PD-L1+ cells between the two groups. Representative mIHC staining images are provided in **Supplementary Figure 1**.

### TIME differences between locoregionally advanced primary and metastatic NPC

**Figure 3:**
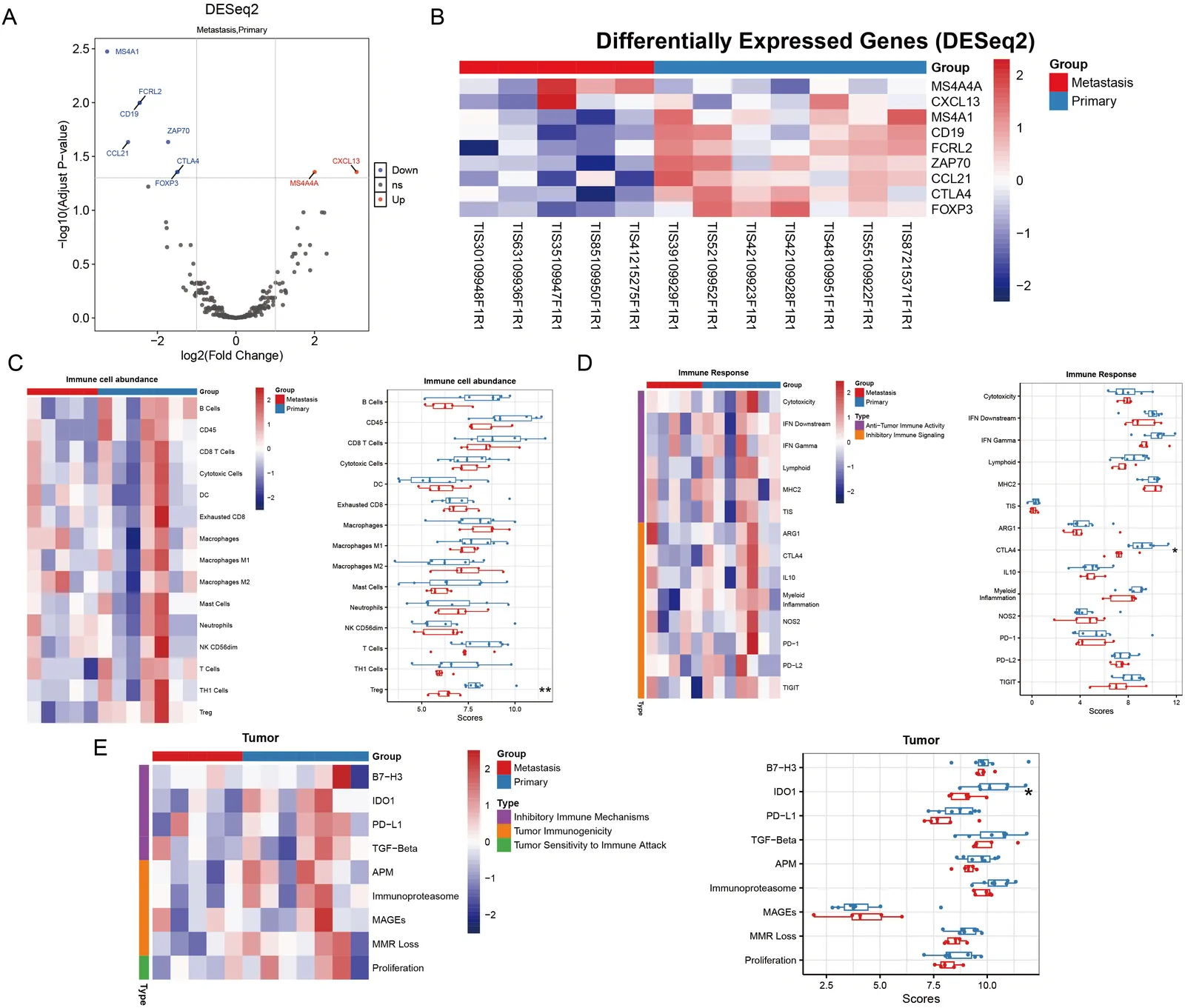
Immune landscape differences between primary and metastatic NPC. **(A, B)** Volcano plot and heatmap showing differentially expressed genes between primary and metastatic NPC tumors identified by NanoString RNA-seq (DESeq2 analysis). **(C, D)** Heatmaps and box plots illustrating immune cell abundance and immune response scores derived from NanoString immune profiling, revealing lower regulatory T cell (Treg) and myeloid-related signatures in metastatic lesions. **(E)** Expression heatmap and box plots of immune checkpoint and regulatory molecules. *P < 0.05; ** < 0.01.

We compared the TIME of primary and metastatic tumors in locoregionally advanced NPC to identify differences in immune cell composition and immune responses (**Figures 3A, B**). Analysis of immune cell abundance revealed significant differences in Treg cells between primary and metastatic tumors. Specifically, primary tumors exhibited a markedly higher abundance of Treg cells compared to metastatic tumors, suggesting the presence of an immunosuppressive microenvironment at primary sites (**Figure 3C**). Furthermore, distinct immune response signatures were observed between the tumor microenvironments of primary and metastatic lesions. The results demonstrated that primary tumors displayed elevated expression of immune checkpoints, including CTLA4, compared to metastatic tumors (**Figure 3D**). Notably, primary tumors showed more pronounced activation of immunosuppressive pathways, with high CTLA4 expression levels being associated with attenuated anti-tumor immune responses, indicating less effective immune reactivity.

Meanwhile, consistent with the results observed previously, IDO1 expression was significantly higher in primary tumors of locally advanced NPC than in metastatic tumors. Conversely, markers such as B7-H3, PD-L1, and TGF-β did not demonstrate significant differences between the two groups (**Figure 3E**). This finding suggests that IDO1 may play a key role in the process of immunosuppression and metastatic progression in primary tumors.

### TIME differences between NPC with liver metastasis and non-liver metastasis

**Figure 4:**
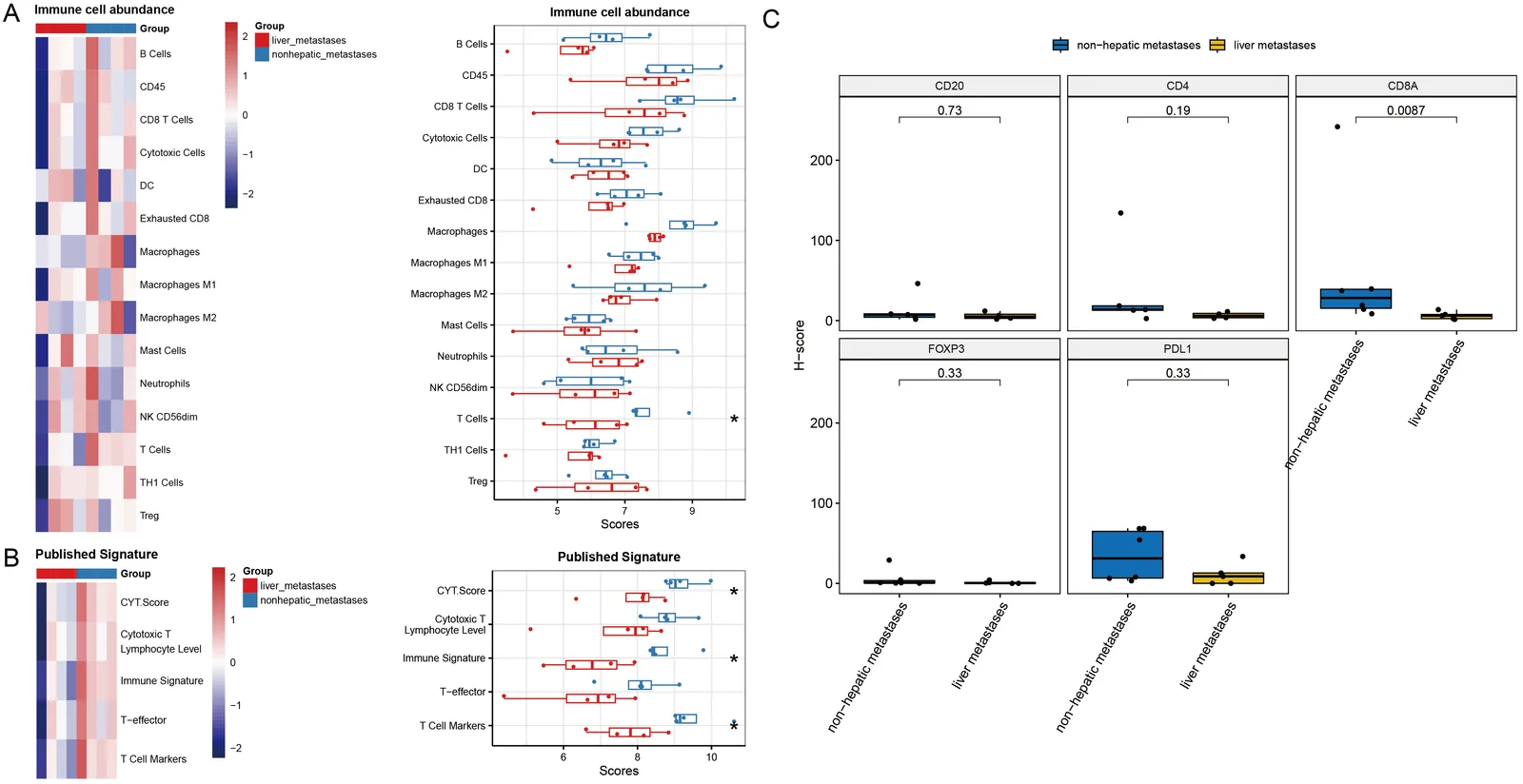
Distinct immune microenvironment between liver and non-liver metastases in NPC. **(A)** Heatmap and box plots showing differential immune cell abundance between liver and non-hepatic metastatic NPC lesions, indicating distinct immune cell infiltration patterns. **(B)** Immune response scores and published immune signatures, including cytotoxicity and T cell-related pathways, demonstrating altered immune activity in liver metastases. **(C)** Multiplex immunohistochemistry (mIHC) analysis comparing CD20, CD4, CD8, FOXP3, and PD-L1 expression between liver and non-liver metastases. *P < 0.05; **< 0.01.

The TIME of liver metastases and non-liver metastases was compared to assess differences in immune cell populations and immune responses. A comparative analysis of immune cell abundance between the two groups revealed that liver metastases exhibited a significantly higher T cell presence compared to non-hepatic metastases (**Figure 4A**), suggesting a more active immune infiltration in the liver metastatic environment. Furthermore, an assessment of immune response markers revealed that liver metastases exhibited elevated scores for CYT (Cytotoxicity), T cell markers, and immune signatures in comparison to non-hepatic metastases (**Figure 4B**). The elevated CYT scores and T cell markers observed in liver metastases imply a heightened immune response in the hepatic microenvironment. These results suggest that liver metastases may have a distinct immune landscape that differs from non-hepatic metastases. Multiplex immunohistochemical analysis of liver metastases and non-liver metastases revealed significant differences in the presence of CD8+ T cells. CD8 expression levels were found to be considerably higher in non-liver metastases compared to liver metastases, indicating a greater abundance of CD8+ T cells in non-liver metastases. In contrast, the expression levels of markers for CD4+ T cells, B cells, and Tregs were comparable in liver metastases and non-hepatic metastases, exhibiting no significant differences (**Figure 4C**). These results imply that the enrichment of CD8+ T cells is distinctly different between liver metastases and non-liver metastases, which may underlie the varied immune responses in these TIME.

### Baseline TIME suggests DFS in advanced localization patients

**Figure 5:**
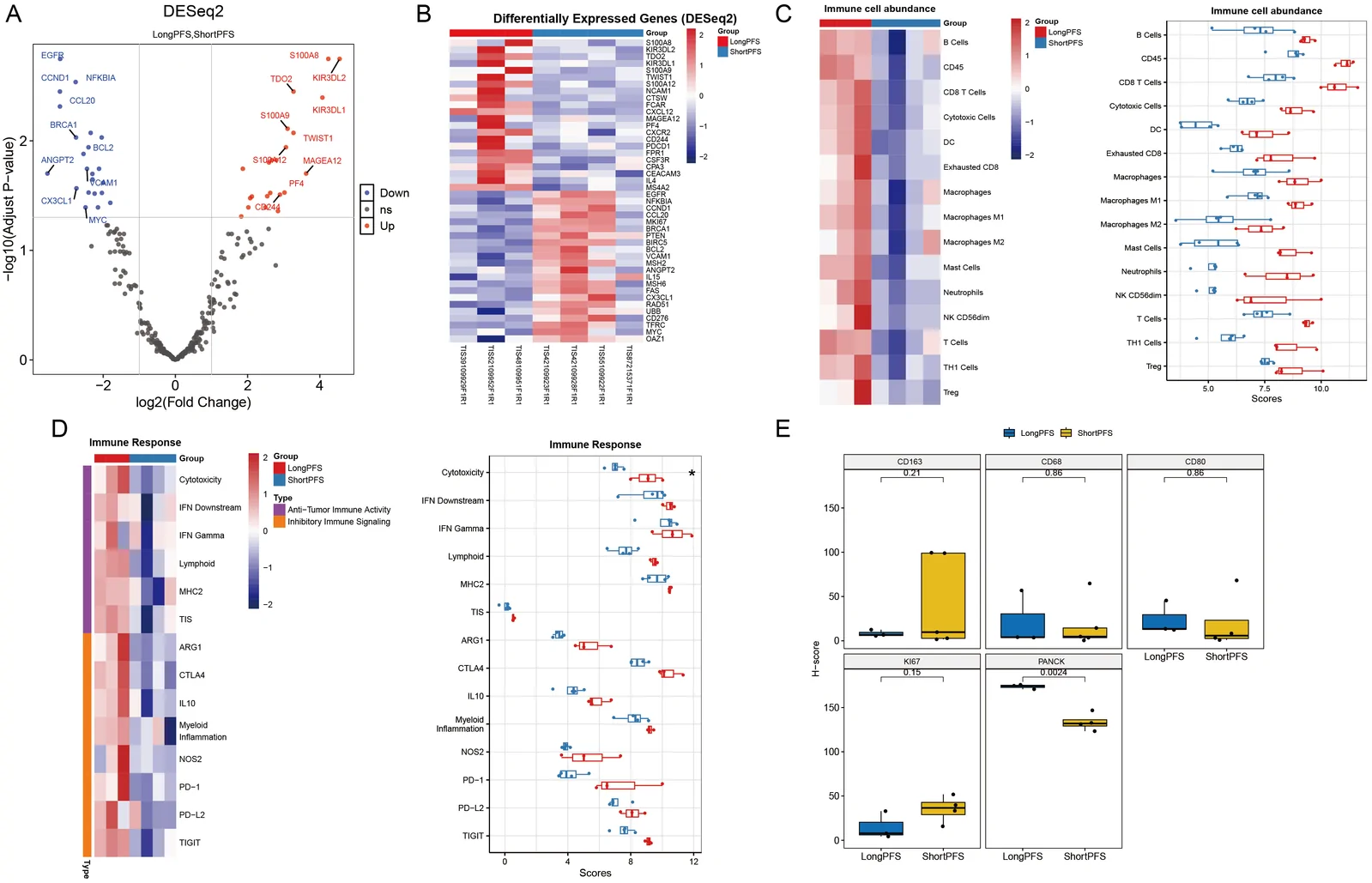
Baseline tumor immune microenvironment associated with disease-free survival (DFS) in locoregionally advanced NPC. Patients were stratified into short DFS (≤20 months) and long DFS (>20 months) groups using a 20-month cutoff, which corresponded to a natural separation of DFS events within the cohort and represented a clinically meaningful threshold for durable disease control. **(A, B)** Volcano plot and heatmap showing differentially expressed genes between long and short DFS groups. Oncogenic genes such as EGFR, MYC, and BCL2 were enriched in the short DFS group. **(C, D)** Heatmaps and box plots illustrating immune cell abundance and immune response scores. The long DFS group exhibited higher cytotoxicity and IFN-γ signaling activity. **(E)** Multiplex immunohistochemistry analysis showing elevated PANCK expression in the long DFS group, whereas the short DFS group exhibited increased macrophage (CD163^+^) infiltration. *P < 0.05; **< 0.01.

In order to investigate the TIME features associated with disease-free survival (DFS) in patients with locoregionally advanced NPC, patients were stratified into short DFS (≤20 months) and long DFS (>20 months) groups. The 20-month cutoff was selected because it corresponded to a natural separation of DFS events within the cohort rather than an arbitrarily chosen threshold. We then performed a differential gene expression analysis comparing patients with short DFS and long DFS. A distinct set of genes was found to be significantly upregulated in the short DFS group, including EGFR, CCND1, NFKBIA, BCL2, and MYC (**Figure 5A**). It is well established that these genes participate in critical oncogenic signaling pathways. Conversely, patients with a longer DFS exhibited reduced expression of these genes, indicating a less aggressive tumour phenotype in baseline.

The investigation revealed that the Long DFS group exhibited significantly higher levels of toxicity scores when compared to the Short DFS group, thereby indicating an enhanced anti-tumour immune response (**Figures 5B, C**). The mIHC results showed that PANCK expression was found to be significantly increased in the Long DFS group (p = 0.0024), indicating greater epithelial differentiation or tumour integrity in these patients (**Figure 5D**). No significant differences were observed in CD68, CD80, CD163, and Ki67, but a trend toward decreased proliferation (Ki67) and macrophage infiltration (CD163) was observed in the Long DFS group (**Figures 5E**).

**Figure 6:**
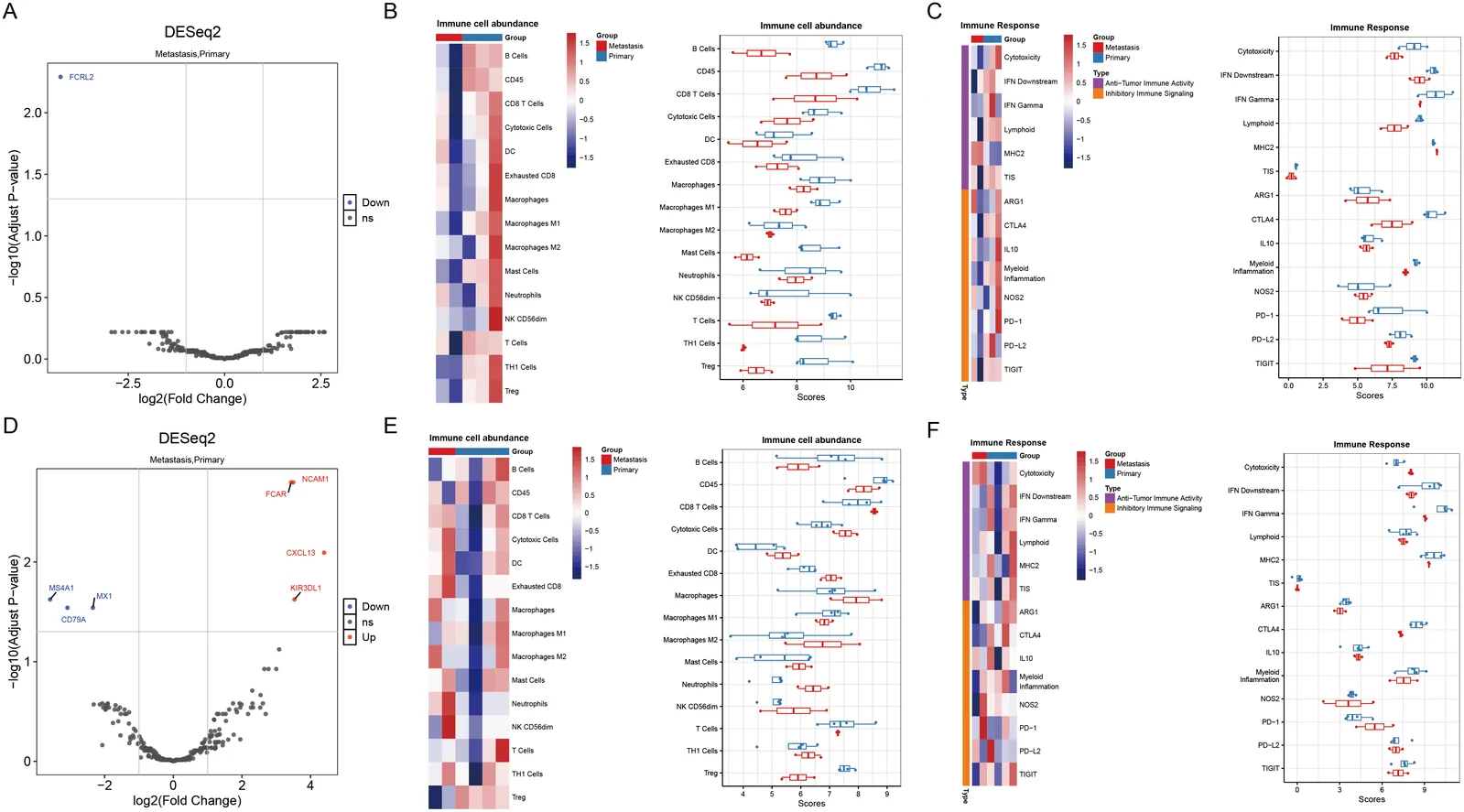
Longitudinal immune microenvironmental changes during metastasis in NPC. **(A, B)** Differential gene expression and immune cell abundance analysis in long DFS patients showing downregulation of FCRL2 and decreased cytotoxic T-cell infiltration in metastatic tumors. **(C)** Immune response profiling indicating reduced cytotoxicity and IFN-γ signaling, along with increased inhibitory markers in metastatic lesions. **(D–F)** In short DFS patients, metastatic tumors exhibited downregulation of B-cell related markers (MS4A1, CD79A) and upregulation of NCAM1, FCAR, CXCL13, and KIR3DL1, reflecting enhanced adhesion, chemokine signaling, and immune evasion. Immune profiling revealed increased macrophage and myeloid-derived suppressor cell-like infiltration in metastases.

To explore the TIME alterations associated with tumor metastasis in patients with locally advanced NPC, we conducted differential gene expression and immune infiltration analyses in short and long DFS groups. In the long DFS cohort, FCRL2 was significantly downregulated in metastatic tumors compared to primary tumors (**Figures 6A, B**). Correspondingly, immune cell abundance analysis showed a downward trend in cytotoxic T cells and increased presence of immunosuppressive cell types such as Tregs and macrophages in metastatic samples. This shift in the immune landscape was corroborated by functional immune response profiling, where metastatic tissues exhibited decreased cytotoxicity and IFN-γ signaling, alongside elevated levels of immune inhibitory markers including ARG1, CTLA4, IL10, and PD-L1 (**Figure 6C**), indicating a transition to an immunosuppressive phenotype during metastasis. In the short DFS cohort, the metastatic tumors exhibited downregulation of B cell–associated markers such as MS4A1 and CD79A, indicating diminished B-cell-mediated responses in the metastatic NPC. Concurrently, upregulation of NCAM1, FACR, CXCL13, and KIR3DL1 was observed in metastases, indicating enhanced cell adhesion, metabolism-related signaling, chemokine-mediated cell trafficking, and NK cell receptor expression, all of which may promote immune evasion and metastatic dissemination. Immune cell profiling of short DFS metastases revealed increased macrophages and myeloid-derived suppressor-like cell populations (**Figures 6D–F**).

## Discussion

NPC is a malignancy with marked geographic distribution, occurring at high frequency in Southern China and Southeast Asia (1). Despite advances in comprehensive treatment, a subset of patients still present with or develop distant metastasis, which is associated with markedly poor prognosis (10). In this study, we performed multi-omics analyses of paired primary tumors and distant metastases, revealing highly similar somatic mutation profiles that suggest metastatic lesions mainly originate from clonal evolution of the primary tumor. In contrast, the immune microenvironment displayed distinct patterns: primary tumors exhibited stronger immunosuppressive features, whereas certain metastatic lesions, particularly liver metastases, showed insufficient immune infiltration. These findings indicate dynamic remodeling of the immune ecosystem during metastatic progression. Furthermore, specific molecular markers and signaling pathways were significantly associated with disease-free survival, suggesting that both molecular alterations and immune contexture jointly influence clinical outcomes. Collectively, our results provide new insights into the mechanisms of NPC metastasis and may inform future strategies for clinical stratification and management.

We found that somatic variations were highly similar between primary and metastatic NPC, with metastatic lesions showing greater enrichment of 1q gain and 6p gain. Chromosomal alterations are common events in NPC, consistent with previous reports. Gains of 1q and 6p have been associated with more advanced disease stages, and focal amplification of 6p, which includes the SOX4 gene, has been linked to lymph node metastasis. No significant survival differences were observed among molecular subgroups defined by molecular characteristics (17, 18). In contrast, our results suggested a trend toward better prognosis in cases with 6p gain, although this did not reach statistical significance. Given the limited sample size, this observation should be interpreted cautiously and may reflect random variation rather than a true biological effect. The apparent discrepancy with previous studies may be attributable to differences in cohort size, patient populations, treatment backgrounds, or analytical strategies. It is noteworthy that previous studies have demonstrated that SOX4 overexpression is associated with cisplatin resistance in NPC (19), whereas lower expression correlates with longer survival. These findings suggest that chromosomal-level alterations may influence disease progression and therapeutic response in NPC. Whether 6p gain influences prognosis through regulation of SOX4 or other genes within this chromosomal region remains unclear and warrants further investigation in larger cohorts.

The tumor microenvironment (TME) of nasopharyngeal carcinoma (NPC) is characterized by prominent immune cell infiltration, including both effector cells (CD4^+^ and CD8^+^ T lymphocytes, B lymphocytes, natural killer cells) and immunosuppressive populations (regulatory T cells [Tregs], tumor-associated macrophages, and myeloid-derived suppressor cells) (20, 21). In our study, primary lesions exhibited a more immunosuppressive phenotype than metastatic sites, characterized by active B-cell infiltration, enrichment of CTLA4^+^ Tregs, and elevated IDO1 expression. This is consistent with previous reports showing that NPC-related factors promote Treg expansion. CTLA4 on Tregs binds CD80/86 on dendritic cells to suppress antigen presentation, while Tregs can also secrete IDO1 to further enhance their proliferation (22). Based on these observations and our findings, we speculate that EBV-associated immune responses, together with increased IDO1 expression, may contribute to the enrichment of CTLA4^+^ Tregs in primary NPC. However, this hypothesis was not directly investigated in the present study, as EBV burden, B-cell receptor repertoire, and spatial interactions between immune cell populations were not evaluated. Therefore, this proposed mechanism should be interpreted as speculative and requires further validation through functional and spatial analyses.

These findings provide direct clinical implications: combining PD-1 inhibitors with CTLA4 or IDO1 blockade is biologically rational. Preclinical studies have shown that anti-CTLA4 therapy can deplete intratumoral Tregs via antibody-dependent cytotoxicity, and subsequent PD-1 blockade significantly increases the frequency of granzyme B^+^CD8^+^and CD4^+^cytotoxic T lymphocytes (23). Clinically, strategies involving ipilimumab in combination or sequence with PD-1/PD-L1 inhibitors have demonstrated higher response rates (~38%) compared to PD-1 monotherapy (20-30%). Notably, in samples that benefited from sequential camrelizumab, intratumoral CD4^+^/CD8^+^T-cell accumulation was observed, further supporting this mechanistic rationale (24–27).

In addition, IDO1 interacts with the PD-1/PD-L1 pathway, as it can directly promote Treg activation through the tryptophan-kynurenine axis. Ongoing clinical trials (NCT03823131) are actively investigating IDO inhibitors in combination with PD-1/PD-L1 blockade or chemotherapy (28). Together, these observations suggest that remodeling the TME through combinatorial strategies may enhance antitumor efficacy. Importantly, our results further imply that such combination approaches might be valuable not only in metastatic NPC but also in treatment-naïve, non-metastatic patients, where early intervention against immunosuppressive mechanisms could potentially improve long-term outcomes.

Our study revealed that liver metastases exhibited a distinct immune-cold phenotype, with markedly reduced T-cell infiltration, particularly CD8^+^T cells, compared to non-hepatic lesions. This finding is consistent with the liver’s intrinsic immune-tolerant nature and may underlie the limited efficacy of immune checkpoint inhibitors in this setting (29, 30). Previous studies have demonstrated that anti-angiogenic therapy can promote vascular normalization improve tumor perfusion, thereby enhancing CD8^+^T-cell recruitment and infiltration (31, 32). Although these mechanisms have not been directly evaluated in NPC liver metastases, they raise the possibility that anti-angiogenic strategies may represent a potential approach to remodel the immune-excluded microenvironment and enhance immunotherapy efficacy in selected patients. This hypothesis requires validation through mechanistic studies and prospective clinical trials.

In addition, we observed that high baseline PANCK expression and enhanced cytotoxicity were associated with longer DFS, whereas activation of PI3K-AKT, and MAPK pathways correlated with shorter DFS. These findings suggest that preserved epithelial differentiation and cytotoxic immune dominance are linked to favorable outcomes, while persistent activation of pro-inflammatory and survival pathways may drive immune escape and early relapse (33, 34). Notably, previous reports in gastric cancer have shown that patients with higher baseline PANCK expression and increased natural killer cell activity also achieved better therapeutic responses (35). In short-DFS patients, metastatic lesions exhibited reduced B-cell responses, upregulation of NK cell-related molecules, and increased infiltration of myeloid suppressive cells, further emphasizing the role of immune imbalance in metastatic progression. These results support the hypothesis that treatment response and prognosis in NPC vary substantially depending on the immune contexture.

This study has several limitations. First, the sample size was relatively small, all enrolled patients were male, and the distribution of metastatic sites was uneven, which may have limited the statistical power and generalizability of our findings. In particular, the non-liver metastatic group comprised several anatomically distinct metastatic sites (including lung, bone, brain, and pelvic metastases), and organ-specific immune heterogeneity may have contributed to the observed differences. Therefore, the comparisons between liver and non-liver metastases should be interpreted as exploratory and require validation in larger cohorts with adequate numbers of each metastatic site to enable organ-specific analyses. Second, although we identified several molecular and immune features associated with metastatic progression, these findings were not validated in an independent external cohort because currently available public nasopharyngeal carcinoma datasets lack paired primary–metastatic samples with comparable longitudinal transcriptomic profiles. Therefore, our findings should be considered exploratory and hypothesis-generating. Third, we were unable to perform functional experiments to investigate the biological mechanisms underlying the identified biomarkers. Future studies integrating paired primary and metastatic specimens, spatial multi-omics technologies, larger multicenter datasets, mechanistic experiments, and emerging computational immune inference frameworks (e.g., CIDE) will be essential to further elucidate metastatic evolution and immune regulation in NPC. Moreover, prospective studies and multicenter clinical investigations are warranted to evaluate the clinical utility of the molecular and immune-based stratification proposed in this study.

In conclusion, this study revealed distinct immune microenvironmental differences between primary and metastatic NPC and their prognostic implications. Primary tumors displayed an immunosuppressive phenotype, suggesting that combining CTLA4 or IDO1 blockade with PD-1 inhibition may enhance therapeutic efficacy. Liver metastases demonstrated an immune-cold profile, supporting the potential benefit of anti-angiogenic therapy in combination with immunotherapy. High PANCK expression and enhanced cytotoxicity were associated with favorable survival, whereas activation of PI3K-AKT, and MAPK pathways indicated a higher risk of recurrence. Overall, our findings provide a biological rationale for future studies aimed at developing stratified and individualized management strategies for NPC.

## Data Availability

The original contributions presented in the study are included in the article/**Supplementary Material**. Further inquiries can be directed to the corresponding author.

## Supplementary material

Supplementary Figure 1 Representative multiplex immunohistochemistry (mIHC) staining images of an NPC tissue sample. **(A)** Merged fluorescence image. **(B–F)** Corresponding single-channel images showing CD8, CD68, CD163, FOXP3, and PD-L1 staining. Images were acquired at ×200 magnification.

## References

[1] Chen YP Chan ATC Le QT Blanchard P Sun Y Ma J . Nasopharyngeal carcinoma. Lancet. (2019) 394:64–80. doi: 10.1016/S0140-6736(19)30956-0 31178151

[2] Chan JK . Virus-associated neoplasms of the nasopharynx and sinonasal tract: diagnostic problems. Mod Pathol. (2017) 30:S68–83. doi: 10.1038/modpathol.2016.189 28060369

[3] Ngan HL Wang L Lo KW Lui VWY . Genomic landscapes of EBV-associated nasopharyngeal carcinoma vs. HPV-associated head and neck cancer. Cancers. (2018) 10:210. doi: 10.3390/cancers10070210 29933636 PMC6070978

[4] Ou SHI Zell JA Ziogas A Anton-Culver H . Epidemiology of nasopharyngeal carcinoma in the United States: improved survival of Chinese patients within the keratinizing squamous cell carcinoma histology. Ann Oncol. (2007) 18:29–35. doi: 10.1093/annonc/mdl320 17060483

[5] Bei JX Zuo XY Liu WS Guo YM Zeng YX . Genetic susceptibility to the endemic form of NPC. Chin Clin Oncol. (2016) 5:15. doi: 10.21037/cco.2016.03.11 27121875

[6] Mao YP Xie FY Liu LZ Sun Y Li L Tang LL . Re-evaluation of 6th edition of AJCC staging system for nasopharyngeal carcinoma and proposed improvement based on magnetic resonance imaging. Int J Radiat Oncol Biol Phys. (2009) 73:1326–34. doi: 10.1016/j.ijrobp.2008.07.062 19153016

[7] Chua ML Zhang X Wong KC Marret G Spreafico A Ma B . Updates on treatments and management of nasopharyngeal carcinoma. Am Soc Clin Oncol Educ Book. (2025) 45:e472460. doi: 10.1200/edbk-25-472460 40209143

[8] Du XJ Wang GY Zhu XD Han YQ Lei F Shen LF . Refining the 8th edition TNM classification for EBV related nasopharyngeal carcinoma. Cancer Cell. (2024) 42:464–473.e3. doi: 10.1016/j.ccell.2023.12.020 38242125

[9] Li WZ Lv SH Liu GY Liang H Guo X Lv X . Development of a prognostic model to identify the suitable definitive radiation therapy candidates in de novo metastatic nasopharyngeal carcinoma: a real-world study. Int J Radiat Oncol. (2021) 109:120–30. doi: 10.1016/j.ijrobp.2020.08.045 32853711 PMC9107935

[10] Co J Mejia MB Dizon JM . Evidence on effectiveness of intensity‐modulated radiotherapy versus 2‐dimensional radiotherapy in the treatment of nasopharyngeal carcinoma: meta‐analysis and a systematic review of the literature. Head Neck. (2016) 38:E2130–42. doi: 10.1002/hed.23977 25546181

[11] Li YY Chung GTY Lui VWY To KF Ma BB Chow C . Exome and genome sequencing of nasopharynx cancer identifies NF-κB pathway activating mutations. Nat Commun. (2017) 8:14121. doi: 10.1038/ncomms14121 28098136 PMC5253631

[12] Bruce JP To KF Lui VWY Chung GTY Chan YY Tsang CM . Whole-genome profiling of nasopharyngeal carcinoma reveals viral-host co-operation in inflammatory NF-κB activation and immune escape. Nat Commun. (2021) 12:4193. doi: 10.1038/s41467-021-24348-6 34234122 PMC8263564

[13] Zhou Z Li P Zhang X Xu J Xu J Yu S . Mutational landscape of nasopharyngeal carcinoma based on targeted next-generation sequencing: implications for predicting clinical outcomes. Mol Med. (2022) 28:55. doi: 10.1186/s10020-022-00479-4 35562651 PMC9107145

[14] Fidler IJ Kripke ML . Genomic analysis of primary tumors does not address the prevalence of metastatic cells in the population. Nat Genet. (2003) 34:23. doi: 10.1038/ng0503-23a 12721548

[15] Liu X Shen H Zhang L Huang W Zhang S Zhang B . Immunotherapy for recurrent or metastatic nasopharyngeal carcinoma. NPJ Precis Oncol. (2024) 8:101. doi: 10.1038/s41698-024-00601-1 38755255 PMC11099100

[16] Liu H Tang L Li Y Xie W Zhang L Tang H . Nasopharyngeal carcinoma: current views on the tumor microenvironment’s impact on drug resistance and clinical outcomes. Mol Cancer. (2024) 23:20. doi: 10.1186/s12943-023-01928-2 38254110 PMC10802008

[17] Wang H Li F Liu N Liu XY Yang XH Guo YM . Prognostic implications of a molecular classifier derived from whole‐exome sequencing in nasopharyngeal carcinoma. Cancer Med. (2019) 8:2705–16. doi: 10.1002/cam4.2146 30950204 PMC6558473

[18] Fang Y Guan XY Guo Y . Analysis of genetic alterations in primary nasopharyngeal carcinoma by comparative genomic hybridization. Available online at: https://onlinelibrary.wiley.com/doi/10.1002/1098-2264(2000)9999:9999<::AID-GCC1086>>.0.CO;2-D (Accessed October 24, 2025). 10.1002/1098-2264(2000)9999:9999<::aid-gcc1086>3.0.co;2-d 11170282

[19] Bissey PA Teng M Law JH Shi W Bruce JP Petit V . MiR-34c downregulation leads to SOX4 overexpression and cisplatin resistance in nasopharyngeal carcinoma. BMC Cancer. (2020) 20:597. doi: 10.1186/s12885-020-07081-z 32586280 PMC7318489

[20] Chen YP Yin JH Li WF Li HJ Chen DP Zhang CJ . Single-cell transcriptomics reveals regulators underlying immune cell diversity and immune subtypes associated with prognosis in nasopharyngeal carcinoma. Cell Res. (2020) 30:1024. doi: 10.1038/s41422-020-0374-x 32686767 PMC7784929

[21] Wang J Luo Y Bi P Lu J Wang F Liu X . Mechanisms of Epstein‐Barr virus nuclear antigen 1 favor Tregs accumulation in nasopharyngeal carcinoma. Cancer Med. (2020) 9:5598–608. doi: 10.1002/cam4.3213 32573058 PMC7402843

[22] Liu Y He S Wang XL Peng W Chen QY Chi DM . Tumour heterogeneity and intercellular networks of nasopharyngeal carcinoma at single cell resolution. Nat Commun. (2021) 12:741. doi: 10.1038/s41467-021-21043-4 33531485 PMC7854640

[23] Harper J Gordon S Chan CN Wang H Lindemuth E Galardi C . CTLA-4 and PD-1 dual blockade induces SIV reactivation without control of rebound after antiretroviral therapy interruption. Nat Med. (2020) 26:519–28. doi: 10.1038/s41591-020-0782-y 32284611 PMC7790171

[24] Lim DWT Kao HF Suteja L Li CH Quah HS Tan DS . Clinical efficacy and biomarker analysis of dual PD-1/CTLA-4 blockade in recurrent/metastatic EBV-associated nasopharyngeal carcinoma. Nat Commun. (2023) 14:2781. doi: 10.1038/s41467-023-38407-7 37188668 PMC10184620

[25] Ma Y Fang W Zhao H Bathena SP Tendolkar A Sheng J . A phase I dose escalation study of the safety, tolerability, and pharmacokinetics of ipilimumab in Chinese patients with select advanced solid tumors. Oncologist. (2021) 26(4):e549–66. doi: 10.1002/onco.13577 PMC8018307 33105036

[26] Fang W Yang Y Ma Y Hong S Lin L He X . Camrelizumab (SHR-1210) alone or in combination with gemcitabine plus cisplatin for nasopharyngeal carcinoma: results from two single-arm, phase 1 trials. Lancet Oncol. (2018) 19:1338–50. doi: 10.1016/S1470-2045(18)30495-9 30213452

[27] Ma Y Zhou H Luo F Zhang Y Zhu C Li W . Remodeling the tumor-immune microenvironment by anti-CTLA4 blockade enhanced subsequent anti-PD-1 efficacy in advanced nasopharyngeal carcinoma. NPJ Precis Oncol. (2024) 8:1–11. doi: 10.1038/s41698-024-00558-1 38448521 PMC10917783

[28] Le Naour J Galluzzi L Zitvogel L Kroemer G Vacchelli E . Trial watch: IDO inhibitors in cancer therapy. Oncoimmunology. (2020) 9:1777625. doi: 10.1080/2162402X.2020.1777625 32934882 PMC7466863

[29] Dara L De Martin E . Immune-mediated liver injury from checkpoint inhibitor: an evolving frontier with emerging challenges. Liver Int Off J Int Assoc Study Liver. (2025) 45:e16198. doi: 10.1111/liv.16198 39868913 PMC11771569

[30] Cao D Zhou A . Tumor immune microenvironment and current status of immune checkpoint inhibitor therapy in colorectal cancer liver metastasis. Curr Oncol Tor Ont. (2025) 32:493. doi: 10.3390/curroncol32090493 41002563 PMC12468209

[31] Song Y Fu Y Xie Q Zhu B Wang J Zhang B . Anti-angiogenic agents in combination with immune checkpoint inhibitors: a promising strategy for cancer treatment. Front Immunol. (2020) 11:1956. doi: 10.3389/fimmu.2020.01956 32983126 PMC7477085

[32] Tang L Hou Y Di T . Anti-angiogenic therapy combined with immune checkpoint blockade mediates CCR7 + CD8 + T-cell entry into HCC through high endothelial venules (2025) (Accessed June 13, 2025). 10.1097/HEP.0000000000001426 PMC13275086 40513067

[33] Fan X Xie X Yang M Wang Y Wu H Deng T . YBX3 mediates the metastasis of nasopharyngeal carcinoma via PI3K/AKT signaling. Front Oncol. (2021) 11:617621. doi: 10.3389/fonc.2021.617621 33816248 PMC8010247

[34] Suryani L Lee HPY Teo WK Chin ZK Loh KS Tay JK . Precision medicine for nasopharyngeal cancer-a review of current prognostic strategies. Cancers. (2024) 16:918. doi: 10.3390/cancers16050918 38473280 PMC10931317

[35] Peng Z Zhang X Liang H Zheng Z Wang Z Liu H . Atezolizumab and trastuzumab plus chemotherapy for ERBB2-positive locally advanced resectable gastric cancer: a randomized clinical trial. JAMA Oncol. (2025) 11:619–24. doi: 10.1001/jamaoncol.2025.0522 40244574 PMC12006909

